# Protoberberine Isoquinoline Alkaloids from *Arcangelisia gusanlung*

**DOI:** 10.3390/molecules190913332

**Published:** 2014-08-29

**Authors:** Ling-Ling Yu, Rong-Tao Li, Yuan-Bao Ai, Wei Liu, Zhang-Shuang Deng, Zhong-Mei Zou

**Affiliations:** 1 Institute of Medicinal Plant Development, Chinese Academy of Medical Science and Peking Union Medical College, Beijing 100193, China; E-Mail: yulingling@ctgu.edu.cn; 2 School of Medicine, China Three Gorges University, Yichang 443002, China; E-Mails: smileday530@163.com (Y.-B.A.); lw2512@ctgu.edu.cn (W.L.); 3 Hainan Branch of the Institute of Medicinal Plant Development, Chinese Academy of Medical Sciences and Peking Union Medical College, Wanning 571533, China; E-Mail: m15572778843@163.com; 4 China Hubei Key Laboratory of Natural Products Research and Development, College of Chemistry and Life Science, China Three Gorges University, Yichang 443002, China; E-Mail: d.zhangshuang@gmail.com

**Keywords:** protoberberine alkaloid, *Arcangelisia gusanlung*, gusanlung E

## Abstract

HPLC**-**DAD-directed isolation and purification of the methanol extract of stems of *Arcangelisia gusanlung* H. S. Lo. led to the isolation of a new protoberberine alkaloid, gusanlung E (**1**), along with fourteen known derivatives **2**–**15**, seven of which were obtained from the genus *Arcangelisia* for the first time. The structures and absolute stereochemistry of these compounds were elucidated on the basis of spectroscopic analyses, including 1D and 2D NMR, mass spectrometry, and CD analyses. Gusanlung E (**1**) expressed weak cytotoxic activity against the SGC 7901 cell line with an IC_50_ value of 85.1 µM.

## 1. Introduction

*Arcangelisia gusanlung* H. S. Lo (Menispermaceae) is a small shrub widely distributed in the south of China including the provinces of Guangdong, Guangxi, and Hainan. The stems of *A. gusanlung* have been clinically used in Chinese folk medicine as an anti-inflammatory, antipyretic, and detoxication reagent [1]. Previous phytochemical investigations of the plant revealed the presence of a series of protoberberine alkaloids [2,3,4] and megastigane glycosides [5] in its stems. Protoberberine alkaloids, which belongs to a isoquinoline alkaloid class, are widely distributed in many species of the Berberidaceae, Annonaceae, Fumariaceae, Papaveraceae, Ranunculaceae, Rutaceae, and other plant families, encompassing a diverse class of secondary metabolites with many pharmacologically active members, such as berberine and palmatine [6,7]. Over the last decade, these alkaloids have attracted considerable attention due to their wide range of biochemical and pharmacological actions, which have applications in various therapeutic areas such as cancer, inflammation, diabetes, depression, hypertension, and various infectious areas [8].

**Figure 1:**
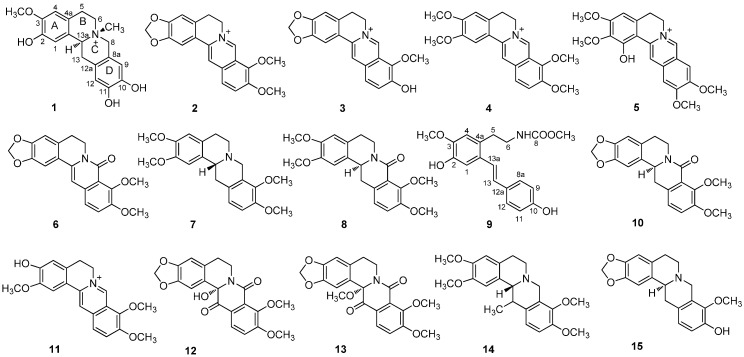
Structures of compounds **1**–**15**.

In order to further investigate the active components of *A. gusanlung*, HPLC-DAD-directed isolation was carried out on the CH_3_OH extract of the stems of *A. gusanlung*. As a result, 15 protoberberine alkaloids including a new one, gusanlung E (**1**), together with fourteen known derivatives **2**–**15**, seven of which were obtained from the genus *Arcangelisia* for the first time (Figure 1). Herein, we report the detailed isolation and structural characterization of these compounds, as well as cytotoxic activity of gusanlung E (**1**).

## 2. Results and Discussion

### 2.1. Structural Characterization

Gusanlung E (**1**) was obtained as yellow crystals, and its molecular formula was determined as C_19_H_22_NO_4_ by HR-ESI-MS at *m/z* 328.1581 [M]^+^ (calcd. for C_19_H_22_NO_4_: 328.1549), indicating ten degrees of unsaturation. The ^1^H-, ^13^C-NMR and HSQC spectroscopic data suggested the presence of 19 carbons. The ^1^H-NMR spectrum showed four aromatic protons at δ 6.86, 6.71, 6.62 and 6.61, one aromatic methoxyl group at δ 3.87 (3H, s) and an *N*-methyl signal at δ 3.20 (3H, s). The signal at δ 3.26 (2H, m) was assigned as H-5, whereas the signals at 3.49 (1H) and 3.82 (1H) were assigned as germinal protons to H-6. Moreover, signals of a pair of methylene protons and an isolated -CH-CH_2_- moiety were found in the aliphatic region. The large coupling constant (15.0 Hz) of a pair of doublets at δ 4.71 and δ 4.52 suggested the existence of germinal protons, which was confirmed by HSQC. This is a typical characteristic of methylene group (C-8) of the protoberberine alkaloids [9]. The signals of an isolated -CH-CH_2_- moiety were assigned to C-13a and C-13. In addition, there were three exchangeable protons were observed at δ 9.13 in the proton NMR spectrum of DMSO-*d*_6_. Analysis of the ^1^H-, ^13^C-, and HSQC NMR spectroscopic data (Table 1) revealed that there were twelve aromatic carbon signals: four aromatic methylene (δ_C_:115.8, 114.5, 114.1, 113.3), eight aromatic quaternary (four oxygenated); four methylene; one methane; one aromatic methoxyl (δ_C_ 57.6) and one *N*-methyl carbon (δ_C_ 50.7). According to the above information, the structure of **1** was closely related to the 2,3,10,11-tetrasubstituted-*N*-methyltetrahydroprotoberberine skeleton [10,11]. The complete assignments were accomplished using ^1^H-^1^H COSY, HSQC, HMBC and NOESY spectra.

**Table 1:** ^1^H- (600 MHz, δ ppm, *J* in Hz), ^13^C-NMR (150 MHz, δ ppm), COSY and HMBC spectroscopic data for compound **1** in methanol-*d*_4_.

Position	δ_C_	δ_H_ (*J* Hz)	COSY	HMBC
1	114.5, CH	6.71 s		C-3, C-4a, C-13a
1a	125.8, C			
2	147.5, C			
3	150.1, C			
4	113.3, CH	6.83 s		C-2, C-3, C-1a, C-4a, C-5
4a	120.3, C			
5	24.3, CH_2_	3.28, 3.23 m	H-6	C-1a, C-4a, C-4
6	53.3, CH_2_	3.82, 3.49 m	H-5	C-4a, C-13a, N-CH_3_, C-8, C-5
8	65.1, CH_2_	4.71, 4.52 d (15)		C-12a, C-8a, C-6, C-9, C-13a, N-CH_3_, C-12a
8a	118.0, C			
9	114.1, CH	6.60 s		C-11, C-12a, C-10, C-8a, C-8
10	146.7, C			
11	147.8, C			
12	115.8, CH	6.83 s		C-11, C-10, C-9, C-8a, C-13
12a	122.0, C			
13	35.4, CH_2_	3.35 dd (4.8, 19.6) 3.02 dd (10.2, 18.0)	H-13a	C-8a, C-1a, C-12,
13a	67.6, CH	4.66 dd (6.6, 10.2)	H-13	C-12a, C-4a, C-1, C-8, C-13
3-OCH_3_	56.7, CH_3_	3.87 s		C-3, C-4, C-2
*N*-CH_3_	50.7, CH_3_	3.20 s		C-13a, C-6, C-8

**Figure 2:**
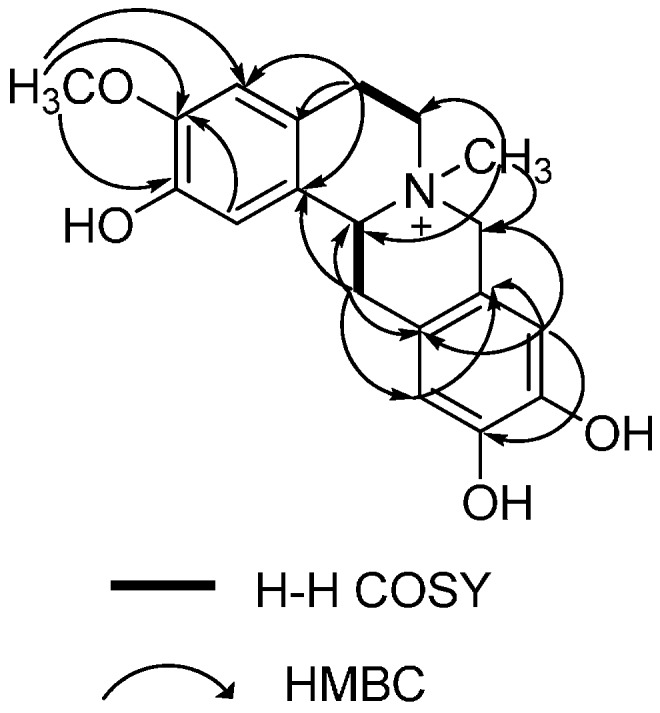
^1^H-^1^H COSY and key HMBC correlations of compound **1**.

**Figure 3:**
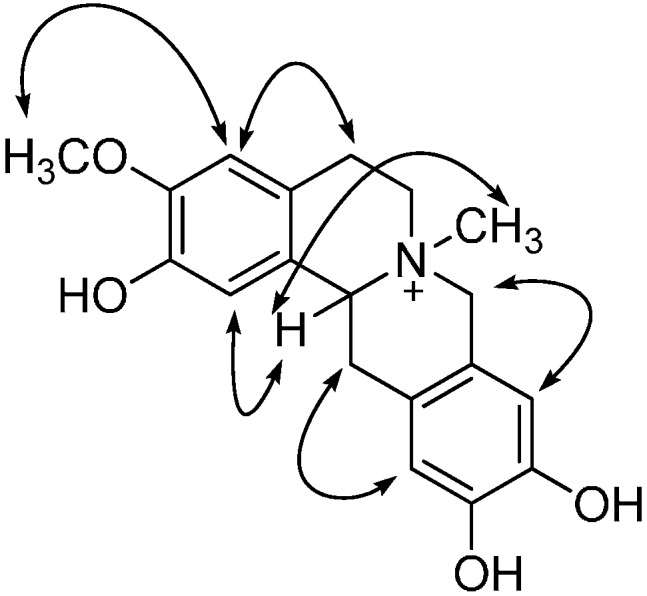
Key NOE of compound **1**.

Interpretation of the ^1^H-^1^H COSY NMR data of **1** confirmed that two isolated proton spin-systems belong to C-5-C-5a and C-13-C-13a units, and the remaining connections were established by analysis of HMBC correlations. The HMBC correlations from -OCH_3_ to C-1, C-3, and C-4, whereas correlations from H-1 to C-3, C-13a and C-4a, and from H-4 to C-1a, C-2 and C-5, indicated that A ring possessed 2-OH and 3-OCH_3_ substitutions (Figure 2). The result was further confirmed by NOESY spectrum, in which the NOE correlations between 3-OCH_3_ and H-4, H-4 and H-5, H-1 and H-13a were observed. In the same way, the cross peaks of H-9 with C-8, C-12a, C-11 and H-12 with C-10, C-8a, C-13 in the HMBC spectrum suggested dihydroxyl substitutions at C-10 and C-11 in D ring. Moreover, H-9 was correlated with H-8 and H-12 with H-13 in the NOESY spectrum (Figure 3). Therefore, the planar structure of **1** was characterized as 2,10,11-trihydroxy-3-methoxy-*N*-methyltetrahydro-protoberberine.

The relative configuration was determined by a NOESY experiment. The *N*-methyl protons showed a NOE correlation with H-13a (Figure 3). Moreover, the NOE correlation between H-6 and *N*-methyl protons suggested the axial position of H-6. The H-13 signal showed a large coupling constant (12.0 Hz) with the signal of H-13a, indicating that H-13 was at axial position [11]. Meanwhile, the ^1^H- and ^13^C-NMR chemical shifts of *N*-methyl group (δ_H_ 3.20, δ_c_ 50.7) as well as a NOESY cross peak between the *N*-methyl group and H-13a suggested a B/C-*cis* fused form [12]. Furthermore, the negative value of specific optical rotation and circular dichroism (CD) curve indicated the 7*S*, 13a*S* configurations [13]. Accordingly, the structure of the new compound was elucidated as shown in Figure 1. 

Compounds **2**–**15** were identified as berberine (**2**) [14], thalifendine (**3**) [15], palmatine (**4**) [16], stephabine (**5**) [17], 8-oxyberbeine (**6**) [18], tetrahydropalmatine (**7**) [19], 8-oxotetrahydroplamatine (**8**) [20], gusanlung C (**9**) [7], gusanlung B (**10**) [6], jatrorrhizine (**11**) [21], 8,13-dioxo-14-hydroxycanadine (**12**) [22], 8,13-dioxo-14-methoxycanadine (**13**) [23,24], corydaline (**14**) [24] and tetrahydrothalifendine (**15**) [25], respectively, by comparison of the ^1^H- and ^13^C-NMR data with reported spectroscopic data. Among them, **5**, **7**, **8**, and **12**–**15** were isolated from this plant for the first time.

### 2.2. Cytotoxic Activities

Gusanlung E (**1**) exhibited weak cytotoxic activity against cell line SGC 7901 with IC_50_ value of 85.1 µM.

## 3. Experimental Section

### 3.1. General Experimental Procedures

Optical rotations were recorded on a JASCO DIP-1000 polarimeter (JASCO, Kyoto, Japan). IR spectra were recorded on a Shimadzu FTIR-8400s (Shimadzu, Kyoto, Japan). UV spectra were run on a Shimadzu UV-2550 UV-VIS spectrophotometer (Shimadzu, Kyoto, Japan). CD spectra were measured on a JASCO J-810 spectrometer (JASCO, Kyoto, Japan). 1D and 2 D NMR spectra were measured in methanol-*d*_4_ (δ_H_ 3.30/δ_C_ 49.5) on a Bruker Avance Ш 600 spectrometer (^1^H: 600 MHz, ^13^C: 150 MHz) (Munich, Ettlingen, Germany). HRESIMS were obtained using a LTQ Orbitrap XL spectrometer (Thermo Fisher, Bremen, Germany). Analytical HPLC was performed on a Waters 600 with a Waters 2996 photodiode array detector (Waters, Milford, MA, USA). Semipreparative HPLC was performed on a Shimadzu LC-6AD with a Shimadzu SPD-6AD spectrophotometric detector (Shimadzu, Kyoto, Japan). 

### 3.2. Plant Material

The stems of *A*. *gusanlung* were collected from Wanning City in Hainan Province of The People’s Republic of China in August 2008. The sample was identified by Prof. Guobiao Chen from the Institute for Drug Control of Hainan Province. A voucher specimen (No. 200808) was deposited in the herbarium of the Institute of Medicinal Plant Development, Chinese Academy of Medical Sciences, Beijing.

### 3.3. Extraction and Isolation

The air-dried and smashed stems of *A*. *gusanlung* (18 kg) were extracted with MeOH (3 × 80 L) and afforded a crude extract of 880 g after evaporation of the solvent under vacuum. The extract was suspended in H_2_O (2.0 L) and partitioned sequentially with petroleum ether (3 × 3.0 L), EtOAc (3 × 3.0 L), and *n*-BuOH (3 × 3.0 L). The EtOAc extract (40 g) was subjected to chromatography over silica gel (800 g, 100–200 mesh) and eluted with CH_2_Cl_2_–MeOH to yield six fractions (E1 to E6) on the basis of TLC and HPLC-DAD analyses. Repeated crystallization of fraction E5 (CH_2_Cl_2_–MeOH) yielded compounds **2** (10 g), **3** (3 g) and **4** (2 g). The *n*-BuOH extract (630 g) was subjected to column chromatography over macroporous resin D101 and eluted successively with EtOH–H_2_O (1:9, 3:7, 6:4, and 1:0) to yield four fractions (B1 to B4). Fraction B1 (10 g) was further subjected to column chromatography over macroporous resin AB-8 and eluted successively with EtOH–H_2_O (1:9, 2:8, 3:7 and 1:0) to yield five fractions (B1-1 to B1-5). Subfraction B1-3 (1.5 g) was subjected to chromatography over silica gel C_18_ (45 g) and eluted with MeOH-Water to yield **1** (50 mg), **14** (7 mg) and **15** (11 mg). Fraction B2 (40 g) was subjected to chromatography over silica gel (400 g, 100–200 mesh) and eluted with CH_2_Cl_2_–MeOH to yield 12 fractions (B2-1 to B2-12) on the basis of TLC and HPLC-DAD analyses. Fraction B2-3 (1.0 g) was subjected to chromatography over silica gel (30 g, 200–300 mesh) and eluted with CH_2_Cl_2_-MeOH to yield 10 subfractions (B2-3-1 to B2-3-10). Subfractions were further separated on Sephadex LH-20 (MeOH) followed by semipreparative HPLC (35% aq. MeOH) to give compounds **5** (8 mg), **6** (23 mg), **7** (20 mg), **8** (12 mg), **9** (10 mg), **10** (27 mg), **11** (12 mg), **12** (120 mg) and **13** (17 mg). 

*Gusanlung E* (**1**): yellow crystals; [α] _D_^20^ −20 (*c* 0.03, MeOH); UV_λmax_ (MeOH) nm 210.0, 287.0; IR_νmax_ (KBr): 3179 (OH), 3042, 2361, 1617 (C=O), 1532 cm^−1^; CD (MeOH) Δε (nm): −9.35 (239), −1.34 (290); ^1^H- and ^13^C-NMR data, see Table 1; HRESIMS *m*/*z* 328.1581 [M]^+^ (calcd for C_19_H_22_NO_4_^+^, 328.1577).

*Berberine* (**2**): yellow crystals; HRESIMS *m/z* 336.1205 [M]^+^ (calcd for C_20_H_18_NO_4_^+^ 336.1236); ^1^H-NMR δ: 3.27 (2H, t, *J* = 6.0 Hz, H-5), 4.11 (3H, s, 10-OCH_3_), 4.21 (3H, s, 9-OCH_3_), 4.94 (2H, t, *J* = 6.0 Hz, H-6), 6.11 (2H, s, -OCH_2_O-), 6.97 (1H, s, H-4), 7.67 (1H, s, H-1), 8.01(1H, d, *J* = 9.0 Hz, H-12), 8.12 (1H, d, *J* = 9.0 Hz, H-11), 8.71 (1H, s, H-13), 9.77 (1H, s, H-8); ^13^C-NMR δ: 26.4 (C-5), 55.2 (C-6), 57.1 (10-OCH_3_), 62.0 (9-OCH_3_), 102.1 (-OCH_2_O-), 105.4 (C-1), 108.4 (C-4), 120.2 (C-13), 120.4 (C-1a), 121.4 (C-8a), 123.5 (C-12), 126.7 (C-11), 130.6 (C-4a), 132.9 (C-12a), 137.4 (C-13a), 143.6 (C-9), 145.4 (C-8), 147.6 (C-2), 149.7 (C-3), 150.4 (C-10).

*Thalifendine* (**3**): faint yellow powder; HRESIMS *m/z* 322.1058 [M]^+^ (calcd for C_19_H_16_NO_4_^+^ 322.1074); ^1^H-NMR δ: 3.24 (2H, t, *J* = 6.0 Hz, H-5), 4.16 (3H, s, 9-OCH_3_), 4.90 (2H, t, *J* = 6.0 Hz, H-6), 6.10 (2H, s, -OCH_2_O-), 6.95 (1H, s, H-4), 7.63 (1H, s, H-1), 7.88 (1H, d, *J* = 9.0 Hz, H-12), 7.78 (1H, d, *J* = 9.0 Hz, H-11), 8.64 (1H, s, H-13), 9.77 (1H, s, H-8); ^13^C-NMR δ: 28.3 (C-5), 57.1 (C-6), 62.4 (9-OCH_3_), 103.6 (-OCH_2_O-), 106.4 (C-1), 109.4 (C-4), 121.7 (C-13), 122.0 (C-1a), 124.4 (C-8a), 124.7 (C-12), 132.4 (C-11), 131.6 (C-4a), 135.3 (C-12a), 139.4 (C-13a), 143.2 (C-9), 145.2 (C-8), 150.0 (C-2), 152.1 (C-3), 150.8 (C-10).

*Palmatine* (**4**): a faint yellow powder; HRESIMS *m/z* 352.1547 [M]^+^ (calcd for C_21_H_2__2_NO_4_^+^ 352.1543); ^1^H-NMR δ: 3.29 (2H, t, *J* = 6.0 Hz, H-5), 3.79 (3H, s, 2-OCH_3_), 3.81 (3H, s, 3-OCH_3_), 3.83 (3H, s, 9-OCH_3_), 3.84 (3H, s, 10-OCH_3_), 4.97 (2H, t, *J* = 6.0 Hz, H-6), 7.01 (1H, s, H-4), 7.66 (1H, s, H-1), 7.97 (1H, d, *J* = 9.0 Hz, H-12), 8.10 (1H, d, *J* = 9.0 Hz, H-11), 8.80 (1H, s, H-13), 9.79 (1H, s, H-8); ^13^C-NMR δ: 27.3 (C-5), 56.6 (C-6), 57.2 (2-OCH_3_), 57.3 (3-OCH_3_), 57.3 (10-OCH_3_), 63.0 (9-OCH_3_), 104.7 (C-1), 110.5 (C-4), 121.5 (C-13), 121.9 (C-1a), 120.1 (C-8a), 124.4 (C-12), 126.9 (C-11), 132.6 (C-4a), 126.9 (C-12a), 138.4 (C-13a), 153.7 (C-9), 145.3 (C-8), 148.9 (C-2), 149.7 (C-3), 144.6 (C-10).

*Stephabine* (**5**): faint yellow powder; HRESIMS *m/z* 368.1486 [M]^+^ (calcd for C_21_H_2__2_NO_5_^+^ 368.1498); ^1^H-NMR δ: 3.24 (2H, t, *J* = 5.4 Hz, H-5), 3.95 (3H, s, 2-OCH_3_), 3.93 (3H, s, 3-OCH_3_), 3.99 (3H, s, 10-OCH_3_), 4.00 (3H, s, 11-OCH_3_), 4.76 (2H, t, *J* = 5.4 Hz, H-6), 6.90 (1H, s, H-4), 7.00 (1H, s, H-12) , 7.62 (1H, s, H-9), 8.84 (1H, s, H-13), 9.14 (1H, s, H-8).

*8-Oxyberberine* (**6**): faint yellow powder; HRESIMS *m/z* 352.1200 [M+H]^+^ (calcd for C_20_H_17_NO_5_ 352.1185); ^1^H-NMR δ: 2.83 (2H, t, *J* = 6.6 Hz, H-5), 3.78 (3H, s, 10-OCH_3_), 3.84 (3H, s, 9-OCH_3_), 3.89 (2H, t, *J* = 6.6 Hz, H-6), 6.01 (2H, s, -OCH_2_O-), 6.69 (1H, s, H-4), 6.95 (1H, s, H-1), 6.53 (1H, d, *J* = 9.0 Hz, H-12), 7.02 (1H, d, *J* = 9.0 Hz, H-11), 7.35 (1H, s, H-13), 8.03 (1H, s, N-H); ^13^C-NMR δ: 29.8 (C-5), 38.8 (C-6), 56.2 (10-OCH_3_), 60.9 (9-OCH_3_), 102.3 (-OCH_2_O-), 103.6 (C-13), 104.9 (C-1), 109.6 (C-4), 109.7 (C-11), 119.7 (C-13a), 124.6 (C-12), 126.4 (C-8a), 129.8 (C-4a), 134.0 (C-12a), 135.9 (C-1a), 148.1 (C-2), 148.8 (C-3), 149.6 (C-10), 153.0 (C-9), 160.3 (C-8).

*Tetrahydropalmatine* (**7**): faint yellow powder; HRESIMS *m/z* 356.1862 [M+H]^+^ (calcd for C_21_H_2__6_NO_4_ 356.1862); ^1^H-NMR δ: 2.68 (2H, m, H-5), 2.84 (1H, dd, *J*_13β,__13a_ = 13.0 Hz, *J*_13β,__13α_ = 15.0 Hz, H-13α), 3.20 (1H, dd, *J*_13α,__13a_ = 3.6 Hz, *J*_13α,__13β_ = 15.6 Hz, H-13β), 3.23 (2H, m, H-6), 3.53 (1H, d, *J* = 15.6 Hz, H-8α), 3.59 (1H, dd, *J*_13a,__13β_ = 12.0 Hz, *J*_13a,__13α_ = 3.6 Hz, H-13a), 3.84 (6H, s, 9-OCH_3_, 10-OCH_3_), 3.86 (3H, s, 2-OCH_3_), 3.88 (3H, s, 3-OCH_3_), 4.26 (1H, d, *J* = 15.6 Hz, H-8β), 6.61 (1H, s, H-4), 6.75 (1H, s, H-1), 7.70 (1H, d, *J* = 9.0 Hz, H-11), 7.85 (1H, d, *J* = 9.0 Hz, H-12 ).

*8-Oxotetrahydroplamatine* (**8**): faint yellow powder; HRESIMS *m/z* 370.2023 [M+H]^+^ (calcd for C_21_H_2__4_NO_5_ 370.1654); ^1^H-NMR δ: 2.78 (1H, dd, *J*_13β,__13a_ = 13.0 Hz, *J*_13β,__13α_ = 15.0 Hz, H-13β), 2.80 (1H, dd, *J*_13α,__13a_ = 3.0 Hz, *J*_13α,__13β_ = 15.0 Hz, H-13α), 2.92 (2H, m, H-5), 3.02 (1H, m, H-6α), 3.90 (9H, s, 3 × OCH_3_), 4.02 (3H, s, OCH_3_), 4.70 (1H, m, H-6β), 5.05 (1H, dd, *J*_13a,__13β_ = 9.0 Hz, *J*_13a,__13α_ = 2.0 Hz, H-13a), 6.67 (1H, s, H-4), 6.68 (1H, s, H-1), 6.95 (1H, d, *J* = 9.0 Hz, H-12 ), 7.00 (1H, d, *J* = 9.0 Hz, H-11); ^13^C-NMR δ: 29.8 (C-5), 38.0 (C-13), 39.2 (C-6), 54.5 (C-13a), 56.2 (3 × OCH_3_), 61.5 (OCH_3_), 109.5 (C-1), 111.5 (C-4), 115.5 (C-11), 120.6 (C-12), 123.0 (C-8a), 127.3 (C-12a), 127.8 (C-4a), 130.7 (C-1a), 147.9 (C-3), 148.0 (C-2), 150.7 (C-10), 153.4 (C-9), 162.7 (C-8).

*Gusanlung C* (**9**): faint yellow powder; HRESIMS *m/z* 314.1395 [M+H]^+^ (calcd for C_18_H_20_NO_4_ 314.1392); ^1^H-NMR δ: 2.70 (2H, t, *J* = 7.2 Hz, H-5), 3.44 (2H, t, *J* = 7.2 Hz, H-6), 3.82 (3H, s, COOCH_3_), 6.46 (1H, d, *J* = 15.6 Hz, H-13a), 6.70 (2H, d, *J* = 8.0 Hz, H-9, H-11), 6.79 (1H, d, *J* = 8.4 Hz, H-4), 6.98 (1H, dd, *J* = 8.4, 2.0 Hz, H-3), 7.01(2H, d, *J* = 8.0 Hz, H-8a, H-12), 7.10(1H, d, *J* = 2.0 Hz, H-1), 7.41 (1H, d, *J* = 15.6 Hz, H-13), 8.00 (1H, t, *J* = 7.2 Hz, H-7); ^13^C-NMR δ: 36.8 (C-5), 40.2 (C-6), 56.5 (COOCH_3_), 111.6 (C-13a), 116.0 (C-8a, C-12), 116.1 (C-4), 119.7 (C-1), 122.5 (C-2), 128.0 (C-1a), 130.3 (C-9, C-11), 131.0 (C-4a), 140.7 (C-13), 148.6 (C-12a), 149.0 (C-10), 156.7 (C-2), 167.0 (C-8).

*Gusanlung B* (**10**): faint yellow powder; HRESIMS *m/z* 353.1252 [M]^+^ (calcd for C_20_H_19_NO_5_ 353.1263); ^1^H-NMR δ: 2.76 (1H, dd, *J*_13β,__13a_ = 13.0 Hz, *J*_13β,__13α_ = 15.0 Hz, H-13β), 2.68 (1H, dd, *J*_13α,__13a_ = 3.0 Hz, *J*_13α,__13β_ = 15.0 Hz, H-13α), 2.83 (2H, m, H-5), 2.97 (1H, m, H-6α), 3.86 (3H, s, 9-OCH_3_), 4.01 (3H, s, 10-OCH_3_), 4.65 (1H, dd, *J*_13a,__13β_ = 13.0 Hz, *J*_13a,__13α_ = 3.0 Hz, H-13a), 4.92 (1H, m, H-6β), 5.96 (2H, s, OCH_2_O), 6.65 (1H, s, H-4), 6.67 (1H, s, H-1), 6.93 (1H, d, *J* = 9.0 Hz, H-12), 7.02 (1H, d, *J* = 9.0 Hz, H-11); ^13^C-NMR δ: 29.0 (C-5), 38.2 (C-13), 39.2 (C-6), 55.5 (C-13a), 56.2 (9-OCH_3_), 61.5 (10-OCH_3_), 101.5 (OCH_2_O), 106.5 (C-1), 108.5 (C-4), 115.8 (C-11), 121.5 (C-12), 125.9 (C-8a), 128.7 (C-4a), 128.9 (C-12a), 131.0 (C-1a), 146.5 (C-2), 146.6 (C-3), 150.1 (C-10), 153.3 (C-9), 162.4 (C-8).

*Jatrorrhizine* (**11**): faint yellow powder; HRESIMS *m/z* 338.1396 [M]^+^ (calcd for C_20_H_20_NO_4_^+^ 338.1392); ^1^H-NMR δ: 3.23 (2H, t, *J* = 6.0 Hz, H-5), 4.04 (3H, s, 2-OCH_3_), 4.18 (3H, s, 9-OCH_3_), 4.15 (3H, s, 10-OCH_3_), 4.95 (2H, t, *J* = 6.0 Hz, H-6), 7.46 (1H, s, H-4), 7.80 (1H, s, H-1), 8.08 (1H, d, *J* = 9.0 Hz, H-12), 8.02 (1H, d, *J* = 9.0 Hz, H-11), 8.81 (1H, s, H-13), 9.70 (1H, s, H-8); ^13^C-NMR δ: 26.8 (C-5), 57.2 (C-6), 56.5 (2-OCH_3_), 62.2 (9-OCH_3_), 115.5 (C-1), 112.5 (C-4), 119.8 (C-13b), 121.5 (C-13), 122.9 (C-12a), 123.0 (C-12), 123.8 (C-11), 130.3 (C-4a), 135.0 (C-8a), 139.4 (C-13a), 144.6 (C-10), 145.3 (C-8), 148.9 (C-2), 149.9 (C-3), 151.7 (C-9).

*8,13-Dioxo-14-hyroxycanadine* (**12**): faint yellow powder; HRESIMS *m/z* 406.0900 [M+Na]^+^ (calcd for C_20_H_17_NO_7_Na 406.0903); ^1^H-NMR δ: 2.97–3.01 (1H, m, H-5a), 3.51–3.55 (1H, m, H-5b), 3.42 (1H, m, H-6a), 3.87 (3H, s, 9-OCH_3_), 3.89 (3H, s, 10-OCH_3_), 4.15 (1H, m, H-6b), 5.96 (2H, s, -OCH_2_O-), 6.67 (1H, s, H-4), 6.81 (1H, s, H-1), 7.29 (1H, d, *J* = 8.4 Hz, H-12), 7.53 (1H, d, *J* = 8.4 Hz, H-11); ^13^C-NMR δ: 31.8 (C-5), 39.5 (C-6), 57.5 (10-OCH_3_), 62.8 (9-OCH_3_), 92.2 (-OCH_2_O-), 103.9 (C-13a), 109.6 (C-12), 110.7 (C-11), 118.8 (C-4), 121.8 (C-1), 124.5 (C-12a), 132.6 (C-8a), 135.4 (C-1a), 138.1 (C-4a), 147.9 (C-2), 148.9 (C-3), 153.4 (C-10), 156.2 (C-9), 168.6 (C-8), 203.9 (C-13).

*8,13-Dioxo-14-methoxycanadine* (**13**): faint yellow powder; HRESIMS *m/z* 397.1158 [M]^+^ (calcd for C_2__1_H_1__9_NO_7_ 397.1162); ^1^H-NMR δ: 2.78–2.80 (2H, m, H-5), 3.16 (3H, s, 14-OCH_3_), 3.21 (1H, m, H-6α), 3.92 (3H, s, 9-OCH_3_), 3.98 (3H, s, 10-OCH_3_), 4.93 (1H, m, H-6β), 5.98 (2H, s, -OCH_2_O-), 6.73 (1H, s, H-4), 6.87 (1H, s, H-1), 7.37 (1H, d, *J* = 9.0 Hz, H-12 ), 7.74 (1H, d, *J* = 9.0 Hz, H-11).

*Corydaline* (**14**): faint yellow powder; HRESIMS *m/z* 370.2011 [M+H]^+^ (calcd for C_22_H_2__8_NO_4_ 370.2018); ^1^H-NMR δ: 0.95 (3H, d, *J* = 7.2 Hz, CH_3_), 2.60 (2H, m, H-5), 3.11 (2H, m, H-6), 3.23 (1H, m, H-13), 3.52 (1H, d, *J* = 15.6 Hz, H-8α), 3.71 (1H, d, *J* = 2.4 Hz, H-13a), 3.89 (12H, m, OCH_3_ × 4), 4.16 (1H, d, *J* =15.6 Hz, H-8β), 6.62 (1H, s, H-4), 6.67 (1H, s, H-1), 6.82 (1H, d, *J* = 9.0 Hz, H-11), 6.90 (1H, d, *J* = 9.0 Hz, H-12); ^13^C-NMR δ: 18.2 (13-CH_3_), 29.2 (C-5), 38.2 (C-13), 51.3 (C-6), 54.0 (C-8), 55.5 (-OCH_3_), 55.6 (-OCH_3_), 56.0 (-OCH_3_), 60.0 (-OCH_3_), 63.0 (C-13a), 108.7 (C-1), 110.8 (C-4), 111.1 (C-11), 123.8 (C-12), 128.4 (C-4a, C-8a, C-1a), 134.8 (C-12a), 146.0 (C-10), 147.1 (C-2), 147.5 (C-3), 145.0 (C-9).

*Tetrahydrothalifendine* (**15**): faint yellow powder; HRESIMS *m/z* 326.1395 [M+H]^+^ (calcd for C_19_H_20_NO_4_ 326.1392); ^1^H-NMR δ: 2.68 (2H, m, H-5), 2.84 (1H, dd, *J*_13β,__13a_ = 12.6 Hz, *J*_13β,__13α_ = 15.6 Hz, H-13α), 3.13 (1H, dd, *J*_13α,__13a_ = 3.6 Hz, *J*_13α,__13β_ = 15.6 Hz, H-13β), 3.32 (2H, m, H-6), 3.53 (1H, d, *J* = 15.6 Hz, H-8α), 3.57(1H, dd, *J*_13a,__13β_ = 12.6 Hz, *J*_13a,__13α_ = 3.6 Hz, H-13a), 3.91(3H, s, OCH_3_), 4.15(1H, d, *J* = 15.6 Hz, H-8β), 5.96 (2H, s, -OCH_2_O-), 6.63 (1H, s, H-4), 6.67 (1H, s, H-1), 6.62 (1H, d, *J* = 9.0 Hz, H-11 ), 6.90 (1H, d, *J* = 9.0 Hz, H-12 ); ^13^C-NMR δ: 29.2 (C-5), 36.2 (C-13), 51.5 (C-6), 53.0 (C-8), 59.0 (C-13a), 55.1 (-OCH_3_), 101.3 (-OCH_2_O-),106.7 (C-1), 110.8 (C-4), 111.3 (C-11), 121.7 (C-12), 125.9 (C-8a), 127.1 (C-12a), 127.8 (C-4a), 131.0 (C-1a), 143.0 (C-10), 144.1 (C-2), 144.0 (C-3), 145.0 (C-9).

### 3.4. Cytotoxicity Testing

The cytotoxicity of the compounds was determined using the colorimetric methylthiazoletetrazolium (MTT) assay with taxol as the positive control (IC_50_ value 0.15 µM). The human stomach cancer cell line SGC 7901 in logarithmic phase were seeded in 96 well flat bottom microtitre plates at a density of 1 × 10^4^ cells per well. cells were washed and maintained with different concentrations of drug, 10 µL MTT was added to the culture medium to a final concentration of 0.5 mg/mL and incubated at 37 °C for 4 h. Formazan crystals dissolved in 100 µL DMSO was added and 10 min later the absorbance of the solution was measured at a wavelength of 570 nm. All assays were carried out in triplicate.

## 4. Conclusions

From the chemical investigation of stems of *A. gusanlung*, fifteen protoberberine alkaloids including a new one, named gusanlung E (**1**), were isolated and identified. Gusanlung E (**1**) showed weak cytotoxicity against cancer cell line SGC 7901. These analogues should be studied in more advanced models to establish *in vivo* efficacy.

## Supplementary Materials

Supplementary materials can be accessed at: http://www.mdpi.com/1420-3049/19/9/13332/s1.

## Supplementary Files

Supplementary File 1
