# Supplementary material for: Protoberberine Isoquinoline Alkaloids from Arcangelisia gusanlung
Source: Molecules. 2014 Aug 29;19(9):13332–41. doi: 10.3390/molecules190913332 (PMC6271463; doi:10.3390/molecules190913332)

Supporting Information

Figure S1. ^{1}H-NMR spectrum of gusanlung E (**1**) in DMSO-d_6 (600 MHz, δ ppm).

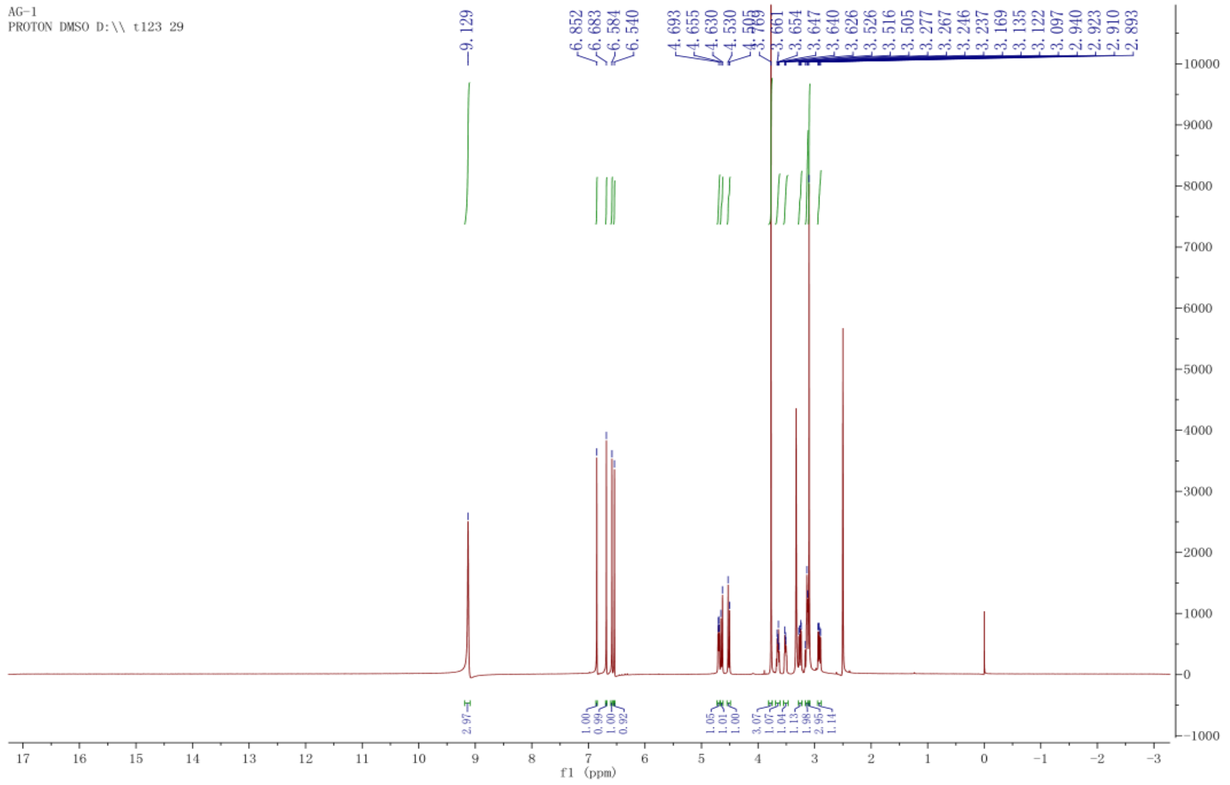

Figure S2. ^{1}H-NMR spectrum of gusanlung E (**1**) in methanol-d_4 (600 MHz, δ ppm).

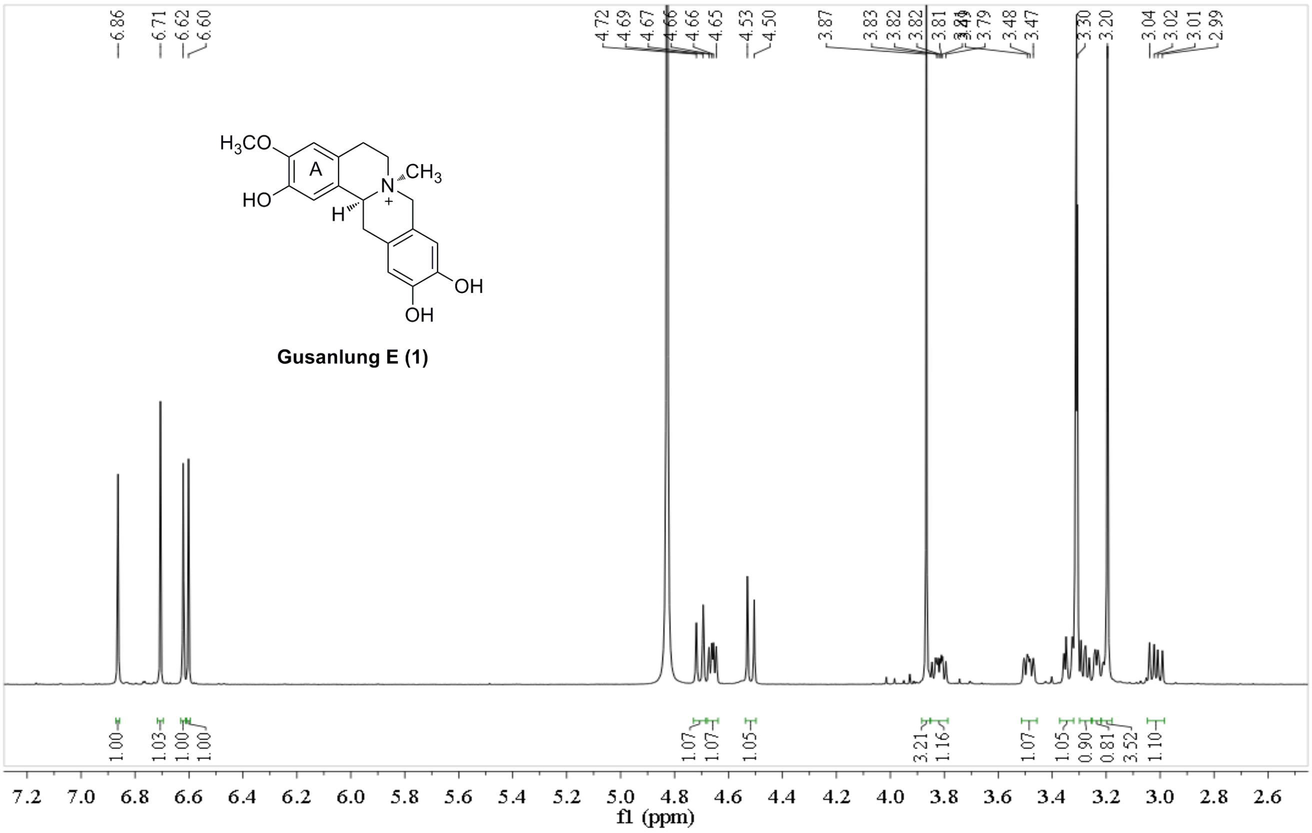

Figure S3. ^{13}C-NMR spectrum of gusanlung E (**1**) in methanol-d_4 (150 MHz, δ ppm).

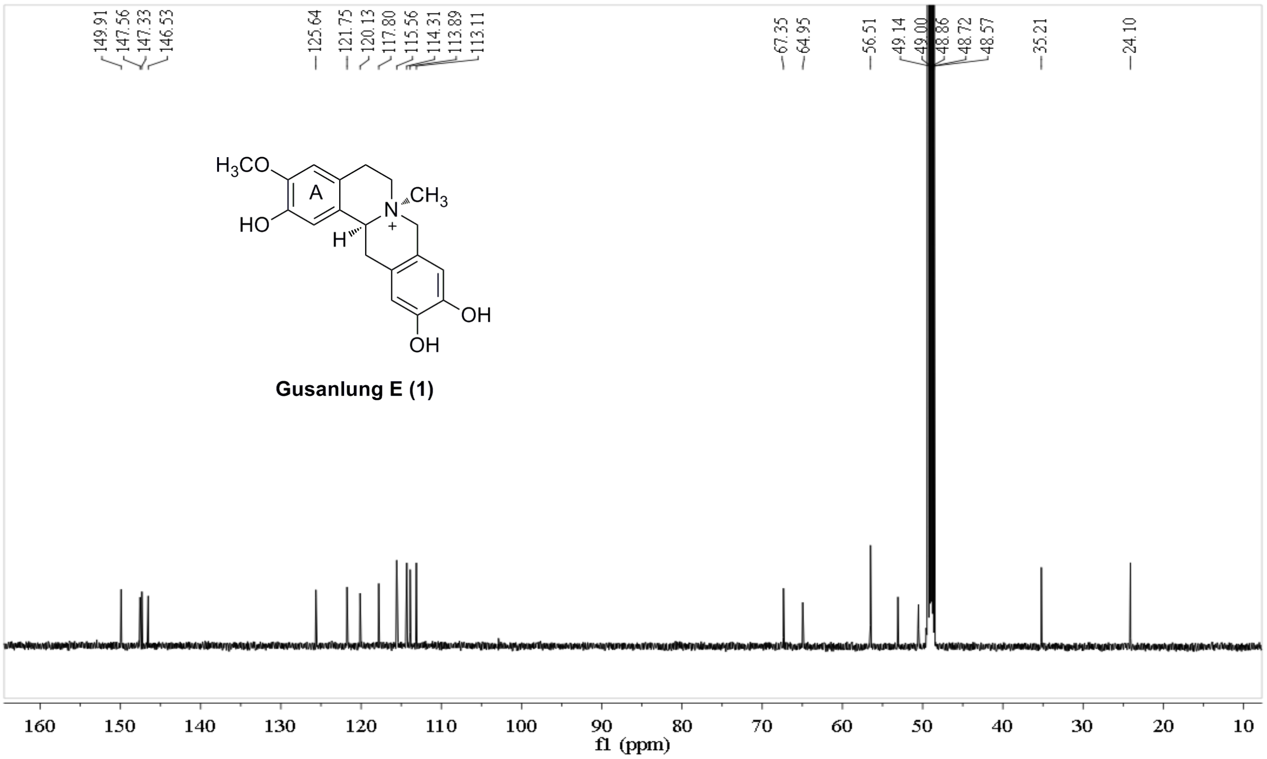

Figure S4. HSQC spectrum of gusanlung E (**1**) in methanol-d_4 (δ ppm).

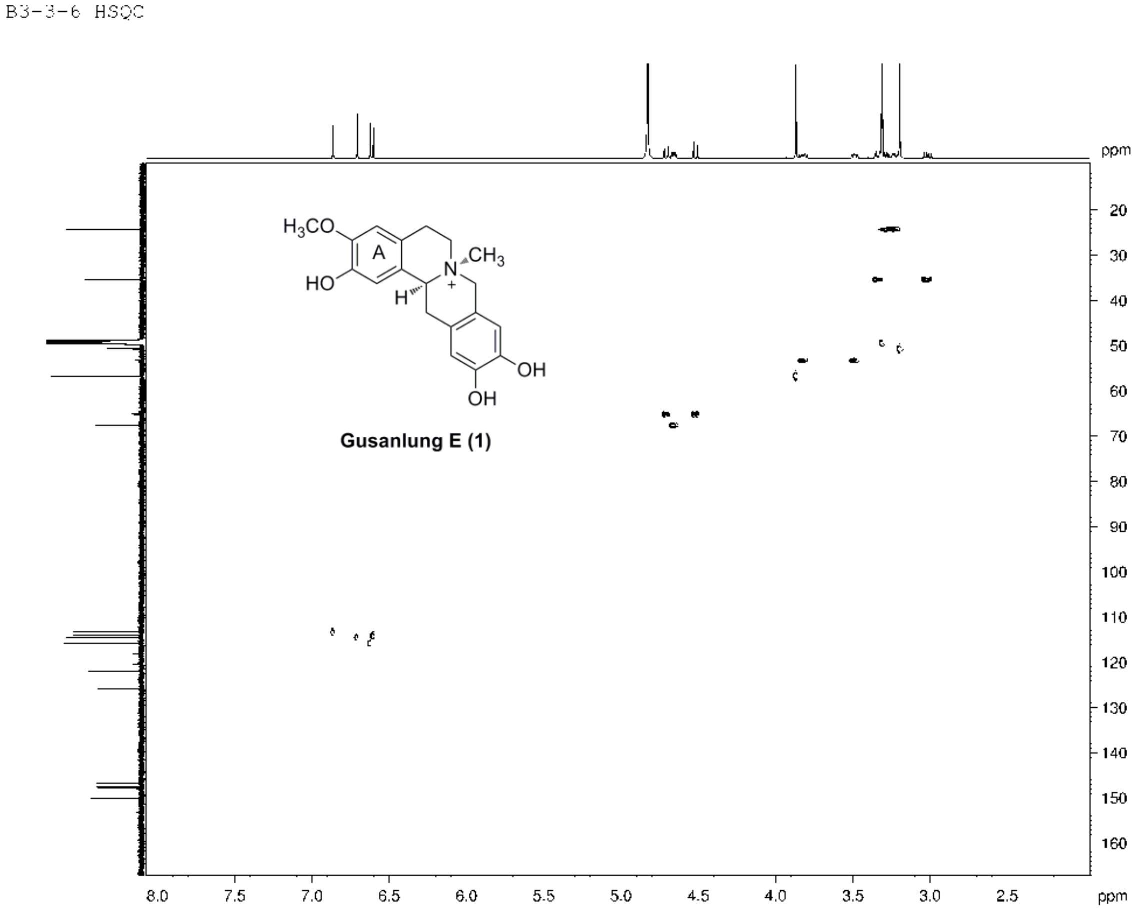

Figure S5. ^1H-^1H COSY spectrum of gusanlung E (**1**) in methanol-d_4 (δ ppm).

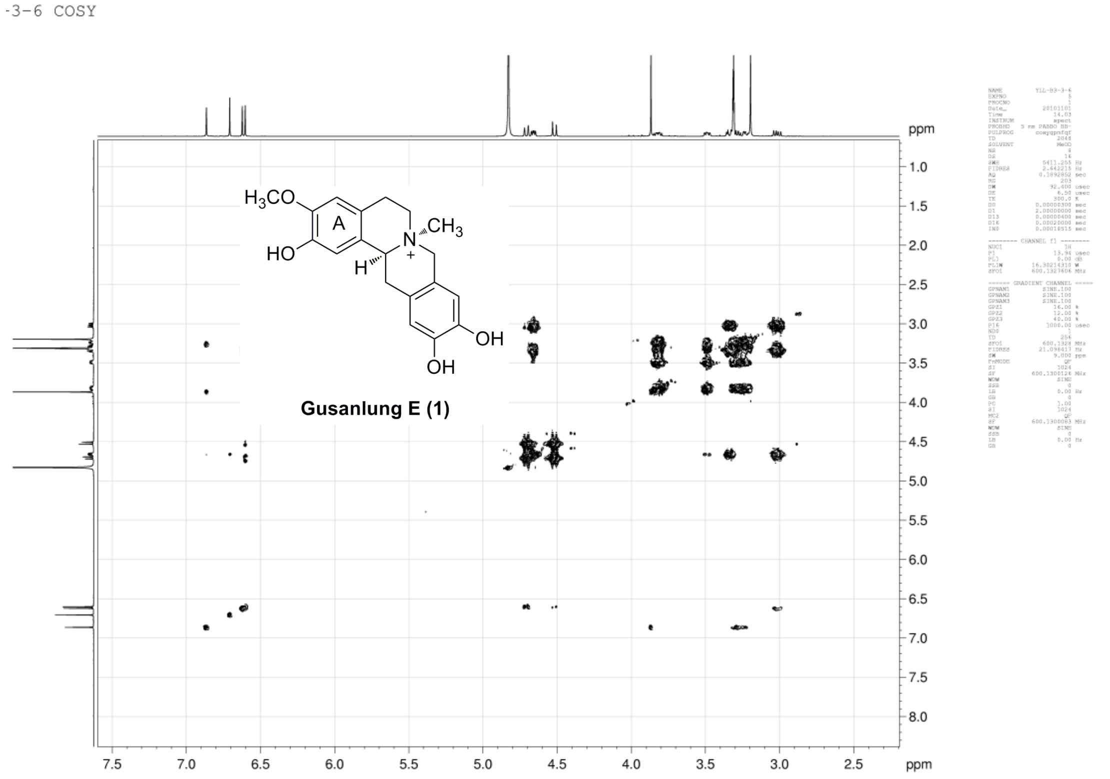

Figure S6. HMBC spectrum of gusanlung E (**1**) in methanol-d_4 (δ ppm).

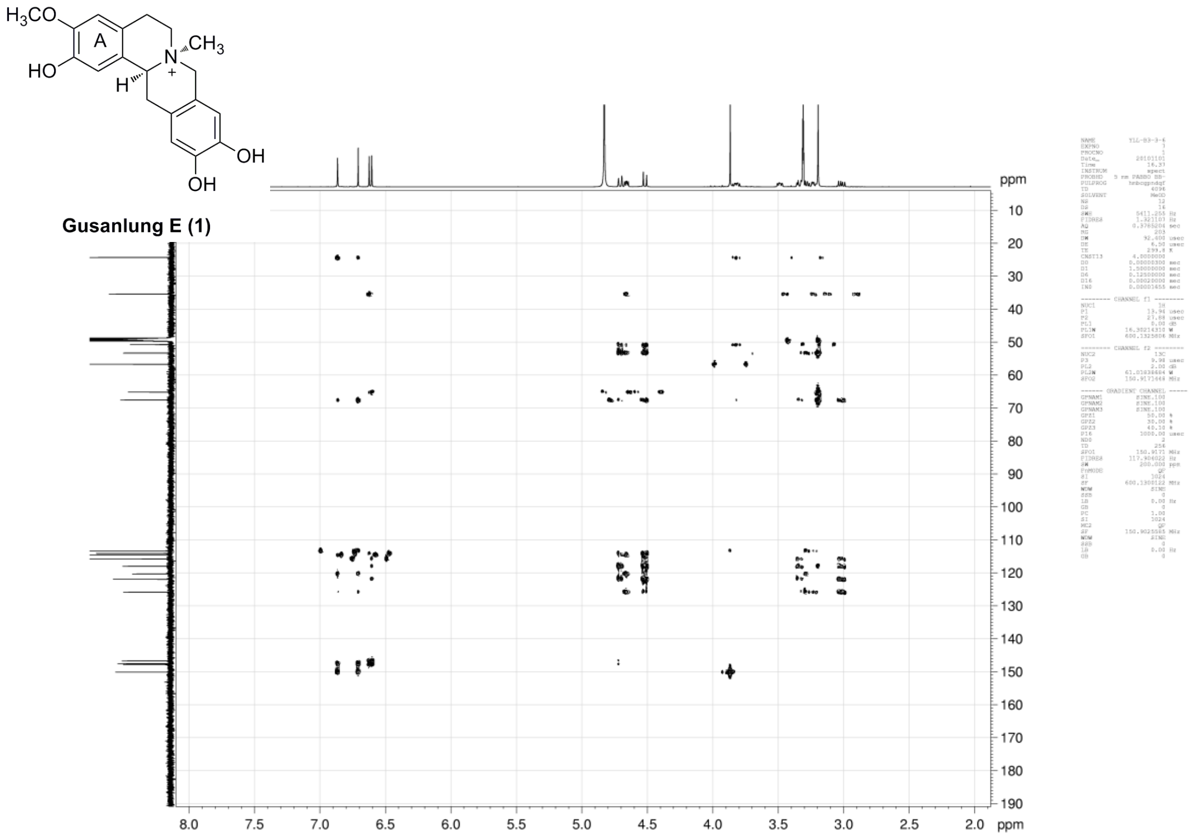

Figure S7. NOESY spectrum of gusanlung E (**1**) in methanol-d_4 (δ ppm).

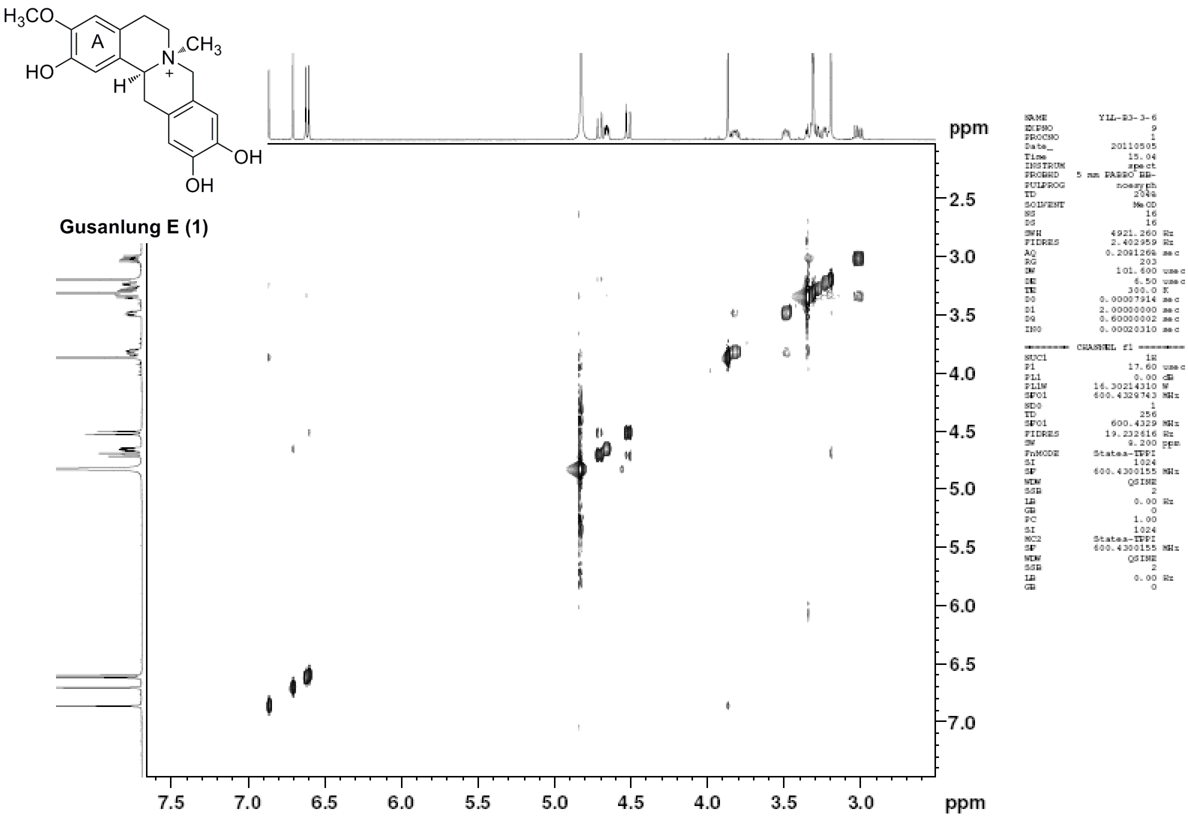

Figure S8. UV spectrum of gusanlung E (**1**) in CH_3OH.

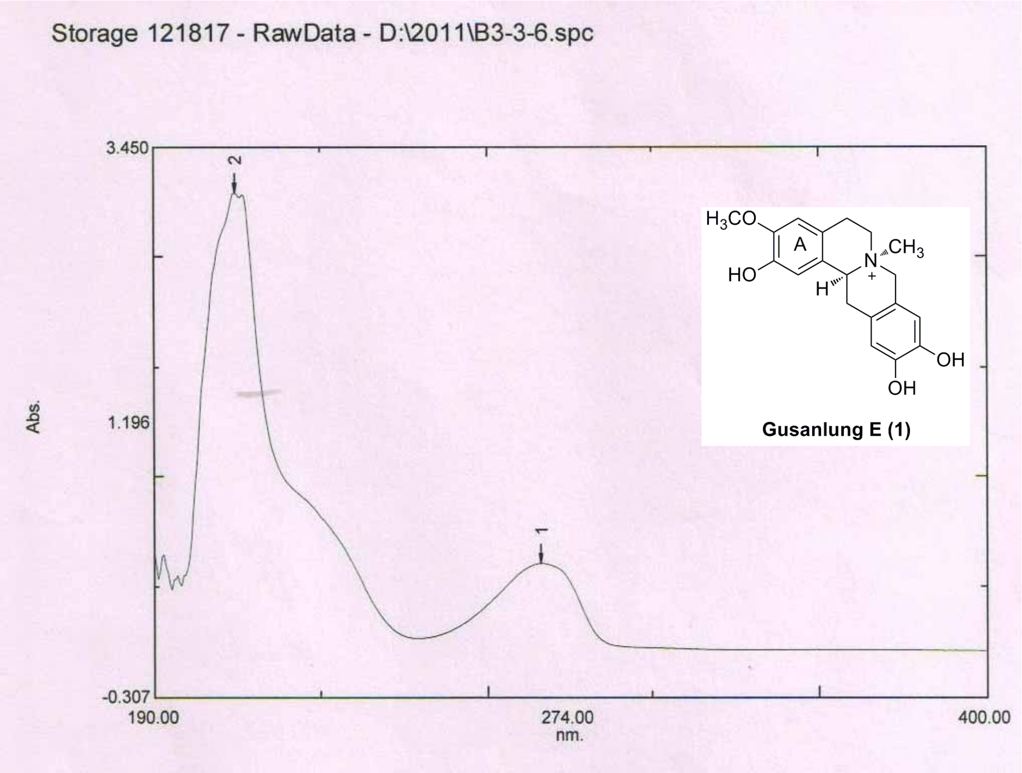

No.	P/V	Wavelength	Abs.	Description
1	⊕	287.50	0.602	
2	⊕	210.50	3.137	

Figure S9. IR spectrum of gusanlung E (**1**).

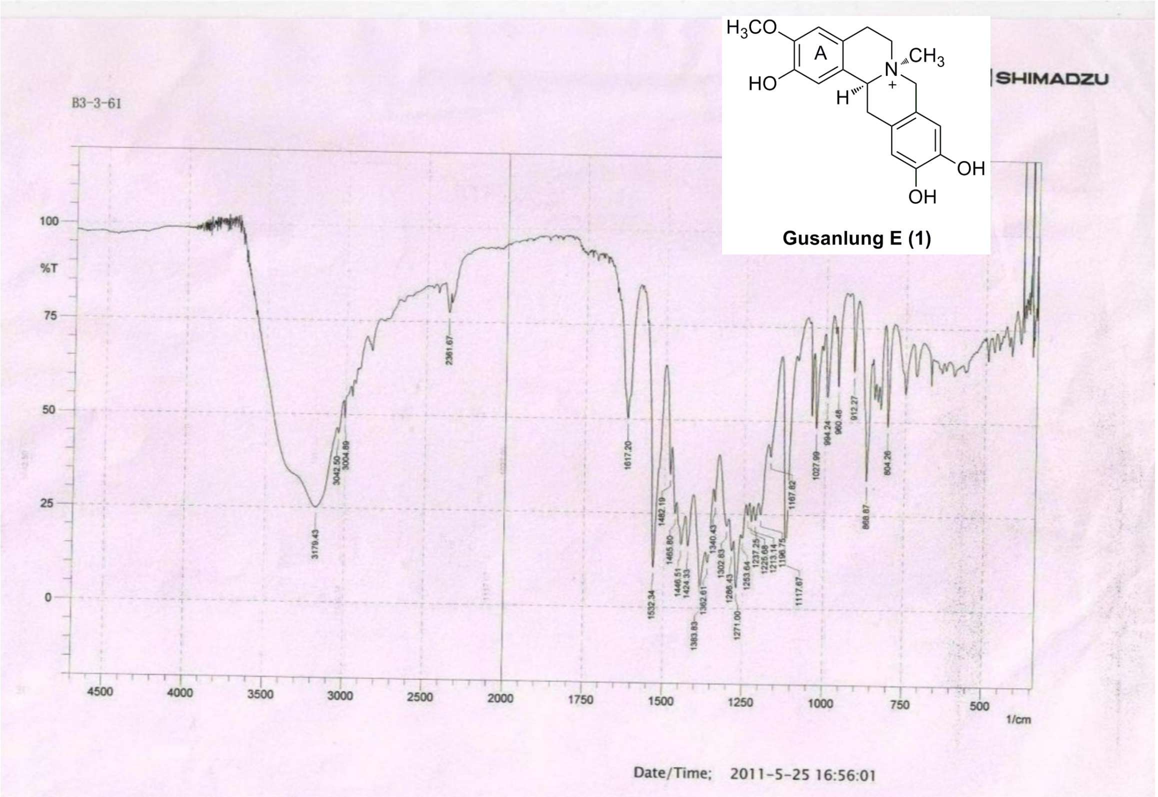

Figure S10. CD spectrum of gusanlung E (**1**) in CH_3OH.

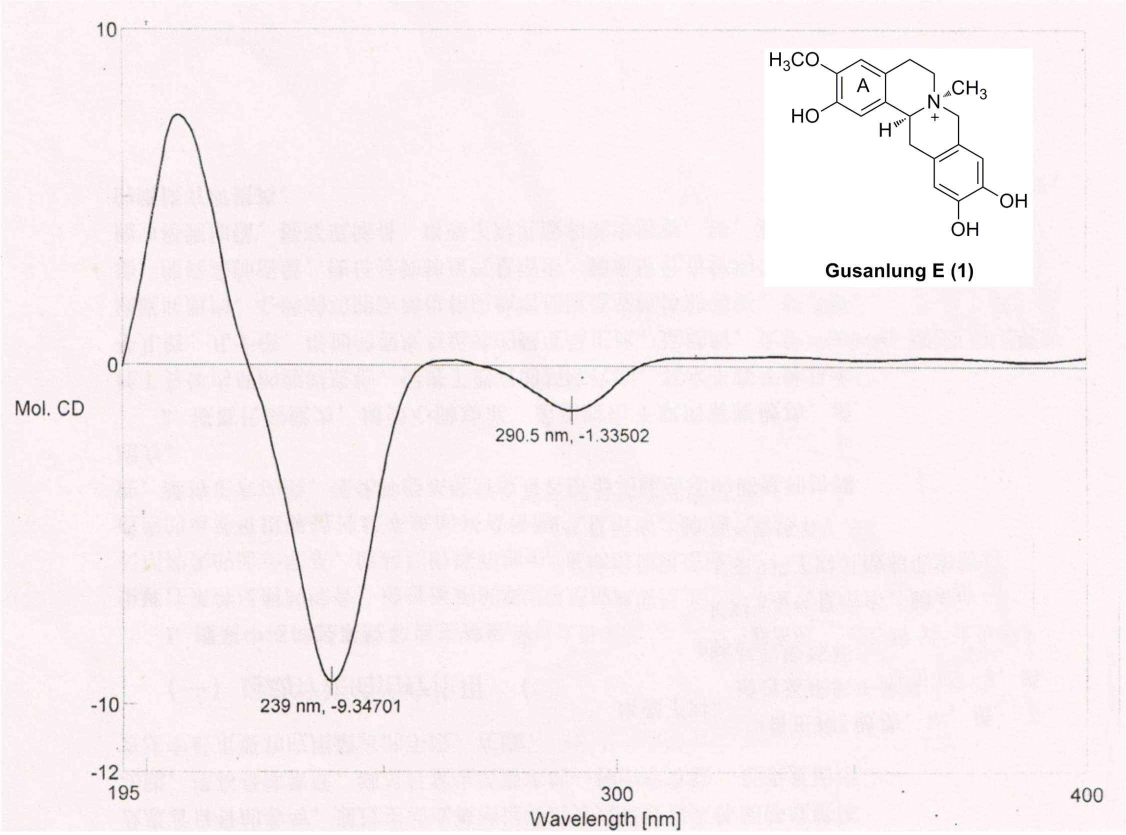

Figure S11. HRESIMS spectrum of gusanlung E (**1**).

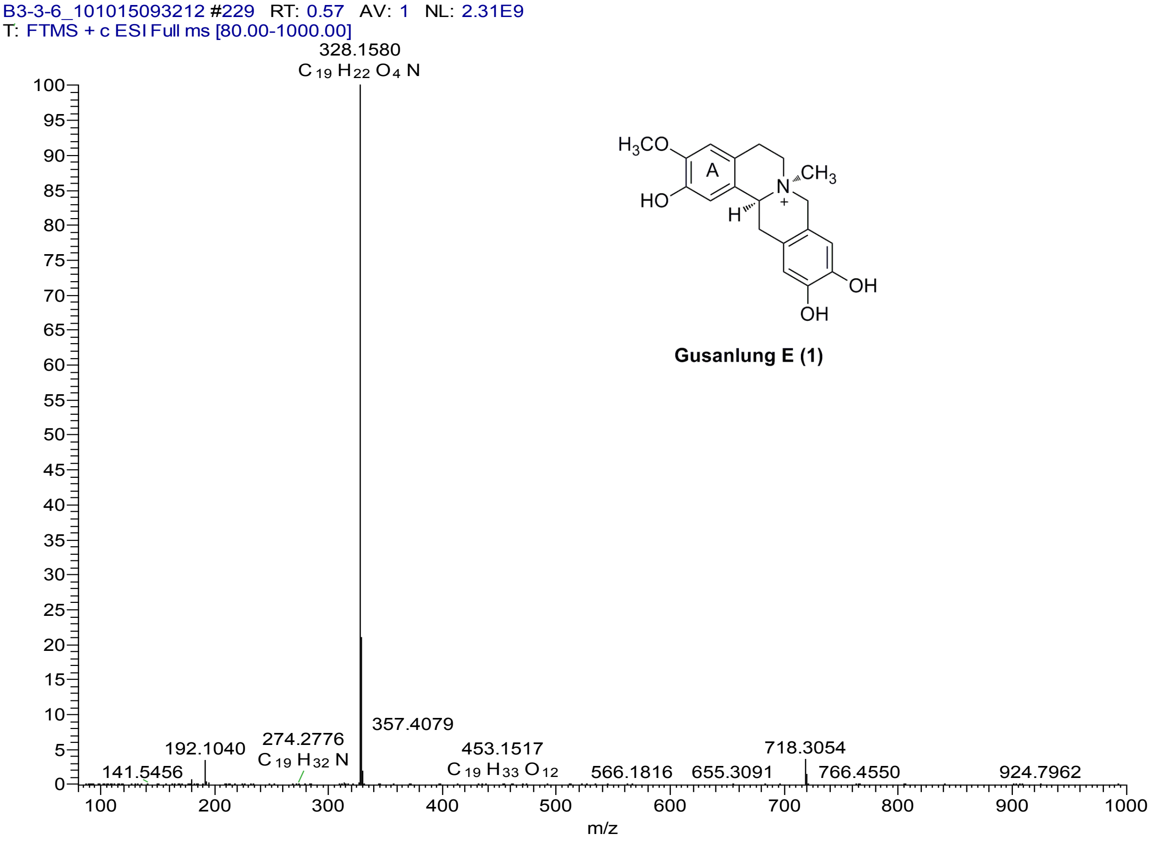

Supplement: Supplementary File 1 [file molecules-19-13332-s001.pdf]
